# High Blood Pressure and Its Association with Body Weight among Children and Adolescents in the United Arab Emirates

**DOI:** 10.1371/journal.pone.0085129

**Published:** 2014-01-20

**Authors:** Abdishakur Abdulle, Abdulla Al-Junaibi, Nicolaas Nagelkerke

**Affiliations:** 1 Department of Internal Medicine, College of Medicine and Health Sciences, UAE University, Al Ain, Abu Dhabi, United Arab Emirates; 2 Department of Pediatrics, Zayed Military Hospital, Abu Dhabi, Abu Dhabi, United Arab Emirates; 3 Institute of Public Health, College of Medicine and Health Sciences, UAE University, Al Ain, Abu Dhabi, United Arab Emirates; Old Dominion University, United States of America

## Abstract

**Objectives:**

To estimate the prevalence of high blood pressure (BP) and its relationship with obesity among children and adolescents.

**Methodology/Principal Findings:**

In this cross-sectional population (Emirati) representative study, we invited a random sample of 1600 students (grades 1–12) attending 23 out of all 246 schools in the Emirate of Abu Dhabi, United Arab Emirates. But analysis was restricted to Emirati nationals aged 6–17 years. We measured BP, height, weight, waist circumferences (WC), and calculated body mass index (BMI) by standard methods. BP levels ≥90^th^ percentile but <95^th^ percentile and ≥ 95^th^ for age, sex, and height (CDC percentiles) were classified as pre-hypertension (pre-HTN) and hypertension (HTN), respectively. Associations between BP, age, BMI, WC, and sex, were investigated by (multiple) linear regression methods. A total of 999 (47% girls) students provided complete results. The prevalence of pre-HTN was 10.5% and 11.4% and the prevalence of HTN was 15.4% and 17.8% among boys and girls, respectively. The prevalence of systolic/diastolic HTN was 14.4%/2.5% and 14.8/7.4% among boys and girls, respectively. BMI CDC percentile was positively correlated with WC percentile (r = 0.734, p<0.01), and both systolic (r = 0.34, p<0.001) and diastolic (r = 0.21, p<0.001) standardized BP. WC percentile was less strongly correlated with standardized SBP (r = 0.255, p<0.01) and DBP (r = 0.175, p<0.01) than BMI.

**Conclusions/Significance:**

The prevalence of elevated BP, notably systolic was significantly high among the Emirati children and adolescents in Abu Dhabi. High BP was strongly related to body weight, and appears more strongly associated with BMI than WC. Further studies are required to investigate the impact of childhood obesity on HTN.

## Introduction

The global burden of diseases and associated risk factors has changed significantly over the past two decades with a clear shift from communicable to non-communicable diseases [1], [2]. Most notably, high blood pressure (BP) has been ranked as the number one contributing factor to the global burden of non-communicable disease, as it is an important and highly prevalent risk factor for both cerebrovascular and cardiovascular disease [3]. Hypertension (HTN) is also increasingly common among children and adolescents, in whom it is sometimes symptomatic and may cause incipient end organ damage [4]–[9].

Childhood overweight and obesity is a main contributing factor to the increasing prevalence of HTN in children and adolescents [3], [10]. Although children with high blood pressure (BP) tend to be heavier than their peers, the best metric for measuring heaviness/obesity is still debated [11]–[13]. As obesity in children is also associated with increased levels of other cardiovascular risk factors, the global childhood obesity epidemic may be the harbinger of a future epidemic of chronic morbidity and premature mortality [14]–[16]. Unfortunately, childhood obesity is highly resistant to simple short-term interventions and often persists into adulthood [17].

HTN in children is, somewhat arbitrarily, defined as the diastolic and/or systolic BP exceeding the 95^th^ percentile for sex, age and height on at least three separate occasions [18]. This dependence on age, sex, and height, however, often makes the diagnosis of HTN in children difficult and therefore often remains undiagnosed [3].

While BP surveys worldwide provide insight into the epidemiology of HTN and its determinants, data interpretation and international comparisons face various methodological limitations including regional disparities in the prevalence and risk factors for HTN [4], [19]. For example, in many formerly resource-poor countries childhood obesity appears to be associated with higher socioeconomic status, in clear contrast with Western countries [20].

Thus, collecting population representative data to estimate the prevalence of HTN in children and its risk factors can strengthen the basis for action to reduce its associated burden of disease and promote health. We therefore carried out a survey among school children aged 6–17 years of Emirati origin in Abu Dhabi Emirate, United Arab Emirates (UAE).

## Subjects and Methods

### Ethics statement

The Al-Ain Medical District Human Research Ethics Committee approved all study protocols and consent forms. Inclusion of students was restricted to those who – together with their parents - gave an informed written consent. For children under 10 years of age only parental consent was sought. Data were collected, revised and pseudonymized by the senior investigator and entered into the study database by trained staff. For ethical reasons, we referred all hypertensive students to pediatric specialists.

### Subjects

A representative sample of Emirati students attending public schools (grade 1–12) were enrolled using a two-stage sampling method; with schools as primary sampling units. Out of 246 schools, 23 were selected. For details on sampling, we refer to an earlier publication [21]. We invited 1600 students of whom 1440 provided complete results (90% response rate), but to avoid potential confounding by ethnicity and adulthood, 405 non- Emiratis and 36 Emiratis adult (>17 years) students were excluded. Thus, analysis was restricted to Emirati nationals aged 6-17 years. Data was collected from January, 2011 to December, 2011 in the Emirate of Abu Dhabi, UAE. Students (if > = 10 years of age) were assisted by their parents to fill out a self-administered questionnaire regarding the health, nutrition and physical activity of the child.

### Anthropometric measurements

In all subjects, qualified nurses who were trained on the study protocols for six hours, weighed the children without shoes or heavy clothing to the nearest 0.1 Kg, and measured the height of the children to the nearest 0.1 cm on a calibrated scale with attached stadiometer (Model 769; Seca, Hamburg, Germany). A standard measuring tape was used to measure waist circumference (WC) at a point right above the iliac crest on the midaxillary line at minimal respiration and was rounded to the nearest 1.0 cm. Three separate measurements of WC, height and weight were recorded for each student and averaged for analysis. Body mass index (BMI), was calculated as the ratio of weight to height squared (kg/m^2^). Children’s weights were classified according to BMI percentile charts for age and sex from the Centers for Disease Control and Prevention (CDC) [22], as underweight: BMI < 5^th^% ile, normal weight: BMI ≥ 5^th^ to <85^th^% ile, overweight: BMI ≥ 85^th^ to < 95^th^% ile, and obese: BMI ≥ 95^th^% ile. WC percentiles were calculated “internally”, i.e., not relative to external standards, using the LMS method (with L,M,S = 3,5,3) [23], which fits smooth curves (splines) to the data after transformation to normality.

### Blood pressure

BP was measured using calibrated Omron M6 IntelliSense (Healthcare, Kyoto, Japan) automatic BP monitors with appropriate cuff size [24]. Prior to taking BP readings, all students were instructed to rest for five minutes, in an air-conditioned environment. Measurements were taken three times on the right arm with short intervals between readings, and the average of the last two BP readings was calculated and used for analysis.

Normotensive: mean SBP or DBP <90^th^ percentile for sex, age, and height; pre-HTN (high normal): mean SBP or DBP levels ≥90^th^ percentile but <95^th^ percentile; HTN: mean SBP or DBP ≥95^th^ percentile for sex, age, and height according to international guidelines [3], [18]. However, we did not measure BP in at least 3 separate occasions (follow up) and therefore the definition of HTN in our study is rather arbitrary. Also, we were unable to distinguish between primary and secondary HTN.

### Statistical analyses

In view of the wide range of variables and comparisons anticipated, we aimed at the largest feasible sample size within our time and financial constraints. Statistical analyses were performed using the Statistical Package for Social Sciences (SPSS) version 19.0 for Windows. Standard univariate and multivariate methods, such as Pearson’s correlation coefficient (r) and multiple linear regression analyses were used. Blood pressure Z-scores were included as the dependent variable, and obesity related percentiles as independent variables, in addition to potential confounders such as age and sex. Standardized scores (“Z-scores”) for BP levels were defined as the difference between observed values and the corresponding CDC 50^th^ percentile, divided by the difference between the 95^th^ and 50^th^ CDC percentile.

A P-value < 0.05 was taken as the statistical significance level.

## Results

A sub-sample of 999 Emirati students under the age of 18 years provided complete information and was available for analysis.

Table 1 shows a summary of subjects’ ages, BMI, and BP. The mean age and SBP was significantly higher among boys than girls, whereas, the mean DBP was significantly higher among girls than boys. The prevalence of pre-HTN was 10.5% and 11.4% and the prevalence of HTN was 15.4% and 17.8% among boys and girls, respectively. HTN among children and adolescents in this population is predominantly ‘systolic’ in nature as shown in Table 2.

**Table 1:** Subject characteristics, by sex.

Parameter	Female (n = 473)	Male (n = 526)	P Value
	Mean (SD)	Mean (SD)	
Age (years)	11.0 (3.4)	11.7 (3.5)	0.002
BMI (kg/m^2^)	20.5 (6.1)	21.2 (7.5)	0.16
BMI percentile (CDC)	60.7 (32.8)	57.6 (34.6)	0.15
SBP (mmHg)	111.2 (10.2)	113.4 (13.1)	0.006
DBP (mmHg)	66.8 (8.0)	65.4 (8.3)	0.007
SBP Z-score	0.47 (0.54)	0.39 (0.57)	(0.021)
DBP Z-score	0.32 (0.44)	0.20 (0.38)	(0.001)

SD; standard deviation, BMI; body mass index, SBP; systolic blood pressure, DBP; diastolic blood pressure, Z-score deviation of BP from age, sex, height specific CDC reference value, divided by the difference between the 95^th^ and 50^th^ CDC percentile.

**Table 2:** The prevalence of pre-hypertension, hypertension, systolic, and diastolic hypertension, by sex, number  =  999 (47% females).

Parameters	Range	Females	Males	Total
		N (%)	N (%)	N (%)
Normal BP	<90^th^	335 (70.8)	390 (74.4)	725 (72.6)
Pre-hypertension (high Normal)	≥90^th^ - <95^th^	54 (11.4)	55 (10.5)	108 (10.9)
High BP (hypertension)	≥95^th^	84 (17.8)	81 (15.4)	165 (16.5)
Systolic hypertension	--	70 (14.8)	76 (14.4)	146 (14.6)
Diastolic hypertension	--	35 (7.4)	13 (2.5)	48 (4.9)

BP; blood pressure.

**Figure 1:**
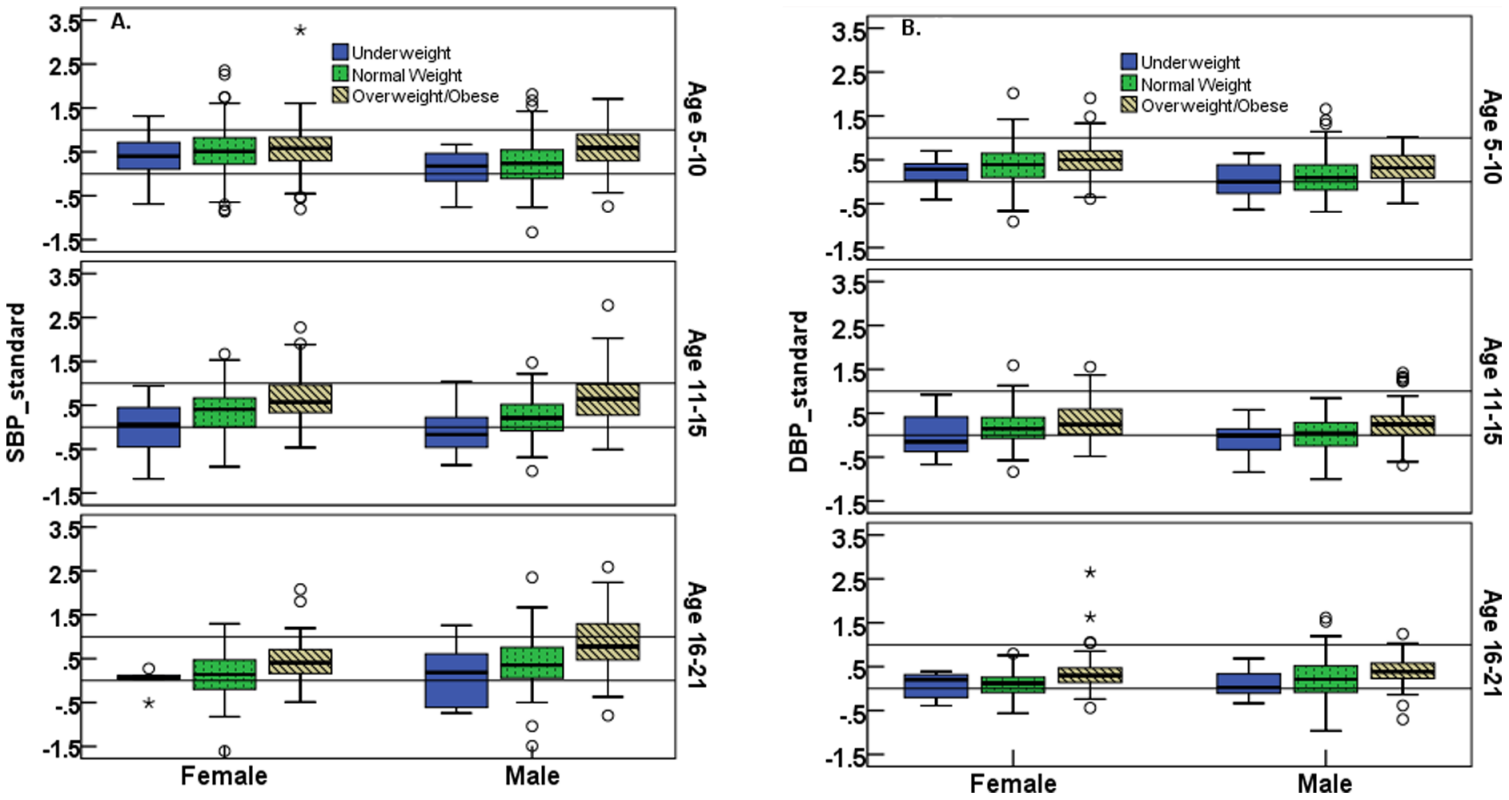
The relationship between blood pressure and children’s weight status. Box plots of standardized systolic (1A) and diastolic (1B) blood pressure (BP), by age group, weight category [underweight (solid boxes), overweight (dotted boxes), obese (line boxes)], and sex. Standardization for age and sex was carried out by subtracting CDC 50^th^ BP percentiles (%ile) from observed values, and dividing this by the difference between the 95^th^ and 50^th^ CDC BP percentile. Thus values > 0 correspond to values above the CDC median (50^th^% ile) and values > 1 correspond to values above the 95^th^% ile, i.e., hypertension.

BMI CDC percentile was positively correlated with WC percentile (r = 0.734, p<0.01), and both systolic (r = 0.34, p<0.001) and diastolic (r = 0.21, p<0.001) standardized BP. WC percentile was less strongly correlated with standardized BP (SBP 0.255, p<0.01; DBP 0.175, p<0.01) than BMI. In stepwise linear regression with age, sex (F/M = 0/1), WC percentile, and BMI CDC percentile (%ile) as candidate independent variables, we found that age [β(se) =  –0.010 (0.005)] and BMI CDC %ile [β(se) = 0.006 (0.001)] were significant predictors (adjusted R^2^  = 0.122) of systolic BP Z-scores, and BMI CDC %ile [β(se) = 0.002 (0.0003)] and sex [β (se) =  –0.113 (0.025)] were significant predictors (adjusted R^2^ = 0.054) of diastolic BP Z-scores. The positive relationship between standardized (i.e., Z-scores) BP levels and weight status (underweight, normal weight, overweight/obese), by age, is shown in Figure 1. It appears that the relationship exists in all age groups and both sexes.

Self-reported chronic disorders were rare in the sample population (diabetes 11, kidney disease 6, heart disease 9, snoring 29, sleep apnea 11, asthma 78) and were not statistically significantly associated with high BP. HTN was reported by three children, of whom only one was identified as hypertensive in our study.

## Discussion

We report a high prevalence of abnormal BP (elevated BPs on one occasion) as pre-HTN (11.0%) or HTN (16.6%) among children and adolescents in the UAE. Evidence shows similarly high prevalence of pre-HTN and HTN despite significant variations at the global level [25]. For example, studies from USA showed a prevalence of pre-HTN (9.5%) and HTN (9.5%) [26]. Comparable results from Switzerland showed a prevalence of 13.3% pre-HTN and 11.4% HTN [19]. Our findings from a single visit “screening” are somewhat higher than found in both the US and Swiss studies.

However, due to logistical reasons, we were unable to collect follow up BP measurements, thus the prevalence levels reported in this population may not represent the actual prevalence of HTN. In addition, while the Omron M6 devise has been calibrated repeatedly, it appears that validation studies specifically in children and adolescents have not been carried out [27]–[29]. Nevertheless, this high prevalence of elevated blood pressure is a finding of great concern; especially since elevated BP (pre-HTN) in children was reported to increase the risk for the development HTN during adolescence [30], as well as the fact that childhood HTN is often a precursor of adulthood HTN and its sequalea. Systolic BP above the 95^th^ percentile among children is known to lead to a more than 4-fold increase in coronary artery disease, compared with SBP below the 95^th^ percentile [14]. Our finding that HTN among children and adolescents in this population is predominantly ‘systolic’ in nature, is therefore of additional concern.

It appears from our study that one of the causes of this problem is the high level of obesity known to elevate the risk for cardiovascular diseases (CVDs) and even all-cause mortality [6]. We previously reported high levels of childhood overweight and obesity among females (34.8%), and males (34.0%) in our sample population [21]. Clearly, interventions targeting childhood obesity should therefore be a priority for public health authorities, especially given the high prevalence of diabetes and other obesity related chronic diseases in the UAE [31].

We report a significantly positive correlation between BMI (CDC percentile) and standardized SBP as well as DBP, though less significantly so. In a stepwise linear regression model, we found that age and BMI were independent predictors of SBP, whereas, sex and BMI were significant predictors of DBP. Although it is difficult to establish unequivocally a causal relationship between obesity and HTN in children from our cross-sectional data, such a relationship seems highly plausible. Interestingly, BMI appeared to be a stronger correlate of BP in this population than WC, unlike findings in China [32]. In our multiple regressions analysis, WC was not even a significant predictor of BP once BMI was taken into account.

Currently, BP measurements are carried out as part of a routine preventive health screening in UAE schools. Nonetheless, the findings at this screening seem to be inadequately interpreted – for example only one of the hypertensive children was aware of his condition - and may thus have little impact.

Taken together, our findings suggest a pressing need for effective methods to address the joint problems of childhood HTN and obesity. In addition to screening and pharmacological interventions, a comprehensive promotion of healthy eating and adequate physical activity may be beneficial [33]. Furthermore, to raise awareness the role of commonly used social networks and social media such as Facebook and Twitter should be explored, taking into account possible cultural idiosyncrasies [34]. Undoubtedly, schools may be used as a proper entry point to implement effective screening and intervention programs. For this, a closer collaboration between educational and health care authorities should be established and the provision of adequate funds should be earmarked for interventional research.

Our study has some strengths and limitations. First, the relatively large sample size and representative nature of our data is of significance and does contribute to the reliability of the outcome. However, one possible limitation is that although we measured BP trice, all measurements took place at one single visit, thus the prevalence of pre-HTN and HTN reported in this study may not necessarily reflect the “true” prevalence of HTN and should therefore be interpreted with caution.

## Conclusion

The prevalence of systolic HTN is significantly high among national children in Abu Dhabi. High BP was significantly related to body weight, though better explained by BMI than WC.
